# Skeletal Muscle Dysfunction and Exercise Intolerance in Children Treated with Haematopoietic Stem Cell Transplant—A Pilot Feasibility Study

**DOI:** 10.3390/ijerph16091608

**Published:** 2019-05-08

**Authors:** Sarah L. West, Gillian White, Jessica E. Caterini, Tammy Rayner, Tal Schechter, Paul C. Nathan, Greg D. Wells

**Affiliations:** 1 Department of Biology, Trent/Fleming School of Nursing, Trent University, Peterborough, ON K9L 0G2, Canada; sarahwest@trentu.ca; 2 Translational Medicine, The Hospital for Sick Children, Toronto, ON M5G 1X8, Canada; gillian.white@sickkids.ca (G.W.); jessica.caterini@sickkids.ca (J.E.C.); 3 Faculty of Kinesiology and Physical Education, University of Toronto, Toronto, ON M5S 1A1, Canada; 4 Radiology, The Hospital for Sick Children, Toronto, ON M5G 1X8, Canada; tammy.rayner@sickkids.ca; 5 Division of Haematology/Oncology, The Hospital for Sick Children, Toronto, ON M5G 1X8, Canada; tal.schechter-finkelstein@sickkids.ca (T.S.); paul.nathan@sickkids.ca (P.C.N.)

**Keywords:** haematopoietic stem cell transplant, pediatric, exercise, muscle metabolism, exercise tolerance, magnetic resonance imaging

## Abstract

Haematopoietic stem cell transplant (HSCT) is an intensive therapy for some pediatric hematological illnesses. Survivors are at risk for adverse effects including exercise intolerance. Peripheral tissue dysfunction may contribute to exercise intolerance; therefore, we examined the feasibility of a magnetic resonance spectroscopy (MRS) protocol to evaluate skeletal muscle metabolism in children post-HSCT. We measured demographic characteristics, aerobic exercise capacity (YMCA protocol), and skeletal muscle function in response to exercise (MRS; Siemens 3T MRI) in five children post-allogeneic HSCT and five age/body mass index-matched healthy controls (HCs). The mean age (± standard deviation) of the HSCT group and HC group were 11 ± 1.2 and 12.8 ± 2.4 years, respectively. Children post-HSCT had a lower peak aerobic exercise capacity compared to HCs (27.8 ± 3.4 vs. 40.3 ± 8.1 mL kg^−1^ min^−1^, respectively; *p* = 0.015). Exercise MRS testing protocols were successfully completed by all HSCT and HC participants; however, MRS-derived skeletal muscle metabolism variables were not different between the two groups. In conclusion, the use of exercise protocols in conjunction with MRS to assess peripheral skeletal muscle metabolism was achievable in children post-HSCT. In the future, larger studies should determine if skeletal muscle function is associated with exercise capacity in children post-HSCT.

## 1. Introduction

Haematopoietic stem cell transplant (HSCT) is a potentially life-saving but high-risk treatment for both malignant and non-malignant pediatric hematological diseases [1]. Allogeneic HSCT requires stem cells from a donor to be transplanted to a patient, effectively replacing the patient’s dysfunctional hematopoietic precursor cells [2]. This treatment is highly intensive, requiring the use of high chemotherapy doses with or without radiation and stem cell infusion. Immunosuppressive therapy is also given to improve engraftment and prevent graft-versus-host disease [3]. While advancements in treatment and expansion of the donor pool have improved survival in children receiving HSCT [1,4,5], many late and long-term adverse effects are associated with this type of treatment. One such adverse effect is exercise intolerance [6,7,8,9,10]. 

Exercise intolerance is common in pediatric HSCT recipients, and manifests as impairments to aerobic function, muscle strength and power, as well as poor balance and motor coordination [11]. These physiological changes can contribute to further physical functional decline, impacting the recipient’s ability to engage in recreational physical activity, resulting in further deconditioning and the reduced ability to perform activities of daily living [11]. This, in turn, may negatively impact the quality of life of HSCT recipients. Furthermore, when compared to conventionally treated childhood cancer survivors (chemotherapy/radiation only), survivors of childhood-HSCT experienced more musculoskeletal impairment and lower physical activity levels [12]. Therefore, HSCT recipients represent a particularly vulnerable group, underscoring the importance of addressing poor exercise tolerance in this cohort.

While exercise intolerance is prevalent in HSCT recipients, the pathophysiology underlying the physical limitations has not been well described. Multiple contributing factors are likely at play. For example, cytotoxic chemotherapy such as cyclophosphamide and total-body irradiation can cause damage to the heart and lungs [13,14,15], which may result in impaired oxygen uptake at the lungs and delivery to tissues for aerobic function. Deconditioning caused by extended periods of inactivity during hospitalization may also lead to exercise intolerance [11]. 

Peripheral tissue dysfunction is another possible factor contributing to exercise intolerance post-HSCT. Children who undergo chemotherapy experience increased systemic inflammation [16,17,18,19,20], which becomes especially apparent following a HSCT in patients presenting with graft-versus-host disease [21]. Systemic inflammation contributes to muscle dysfunction via free radical damage to mitochondria [22,23], resulting in impaired oxidative metabolism [24,25], which can result in impaired muscle function. Once it is determined how skeletal muscle is affected in children post-HSCT, we can develop an informed exercise program to reduce physical morbidity targeting the appropriate energy systems (i.e., anaerobic, aerobic, or a combination of both). However, skeletal muscle metabolism has not been examined in children post-HSCT, due to the invasiveness of research protocols such as muscle biopsies.

Our research team has examined changes to skeletal muscle metabolism in pediatric cohorts with chronic disease by using magnetic resonance spectroscopy (MRS) in conjunction with specific exercise protocols. We have successfully examined skeletal muscle metabolism in children with Turner syndrome, cystic fibrosis, primary ciliary dyskinesia, malignant hyperthermia susceptibility, and obesity/metabolic syndrome [24,26,27,28]. However, we have not piloted the use of exercise MRS assessments in children post-HSCT.

The primary aim of this study was to examine the feasibility of our MRS protocol to evaluate skeletal muscle metabolism in children post-HSCT. We conducted an implementation-focused feasibility pilot study to examine the success or failure of the execution of MRS skeletal muscle testing in children post-HSCT [29]. More specifically, we evaluated skeletal muscle metabolism in response to exercise in five children post allogeneic-HSCT vs. five healthy age and body mass index (BMI) controls (HC). We hypothesized that our protocol would be feasible, i.e., that children post-HSCT would have success in completing the MRS protocol and produce analyzable data [29]. Our secondary aim was to examine if there were any differences in exercise capacity, daily physical activity, or skeletal muscle function post-allogeneic HSCT. We hypothesized that children post-HSCT would have impaired aerobic capacity, poor daily activity levels, and impaired skeletal muscle metabolism compared to the HC group.

## 2. Materials and Methods

### 2.1. Study Participants

Children and adolescents aged 8–18 years who had completed their first allogeneic HSCT within the last 6–36 months at SickKids Hospital were recruited for the HSCT study group. Patients were excluded if they reported any contraindication to exercise and/or magnetic resonance imaging (MRI; i.e., non-compatible implanted device), or body mass >100 kg. All participants in the HSCT group received permission to participate from their primary HSCT physician. Healthy controls (who had no chronic diseases and were generally healthy) were recruited via poster or by word of mouth, and were included based on age and BMI matched to the HSCT group. All study testing occurred at the Hospital for Sick Children in Toronto, and this study was approved by the local research ethics board (REB# 1000039644). All participants were informed about the benefits and risks of the study and provided informed consent to participate in the study.

### 2.2. Descriptive Characteristics and Exercise Tolerance

Height and weight were measured to the nearest 0.1 cm and 0.1 kg, respectively, using a stadiometer (model 555; SR Instruments, Tonawanda, NY, USA). Body composition was measured non-invasively using the BOD POD (COSMED, Rome, Italy). Participants also completed the Habitual Activity Estimation Scale questionnaire [30] to estimate daily physical activity levels.

### 2.3. Systemic Aerobic Capacity

Peak aerobic exercise capacity (VO_2_ peak) was estimated using a sub-maximal cycling test (YMCA protocol) [31]. This cycling protocol involves 3-minute bouts of incremental workloads until approximately 85% of age-predicted maximum heart rate (220-age) is achieved. Heart rate was measured using a Polar heart rate monitor (H7, Polar, Quebec, QC, Canada). Maximum oxygen consumption was determined using standardized calculations. Equation (1) was used to calculate the VO_2_ at respective work rates and subsequently used to find predicted VO_2_ peak using Equation (2) [31].

VO_2_ (mL kg^−1^ min^−1^) = [(10.8 × work rate in watts) / body weight in kg] + 7 (1)

VO_2_ peak (mL kg^−1^ min^−1^) = VO_2_ at work rate 2 + [((VO_2_ at work rate 2 − VO_2_ at work rate 1)/HR_2_ − HR_1_)) × HR_max_ − HR_2_] (2)

### 2.4. Magnetic Resonance Imaging and Spectroscopy

All MRI and spectroscopy data were collected using a Siemens Magnetom Tim Trio^TM^ 3 Tesla MRI (Siemens AG, Medical Solutions, Erlangen, Germany). A dual tuned ^1^H/^31^P transmit/receive surface coil (^1^H: butterfly coil, 180 mm × 244 mm; ^31^P: loop coil, 110 mm diameter) was used for imaging and spectroscopy acquisition. Patients were placed supine and head first inside the magnet. First, T1-weighted anatomical images were acquired axially from the mid-quadriceps region (turbo spin echo (TSE, 2 turbo factor), 200 echo trains per slice (150° flip angle), 10 slices (0.5 mm gap), 5 mm thick, FOV 220 mm, TE/TR = 16/600 ms, total acquisition time = 2 minutes). Following anatomical imaging, with a participant remaining in the scanner, ^31^P-MRS scanning was performed with the same coil.

^31^P-MRS FID spectra were obtained from the vastus lateralis under partially saturated conditions with a hard pulse (0.25 ms duration, 40° flip angle, TR = 1000 ms), eight averages (total acquisition time = 8 seconds per spectrum). Ten resting spectra were acquired and then averaged to determine baseline high energy phosphate content. Subsequently, participants performed a leg extension exercise using an up-down ergometer with power meter (Lode BV Medical Technology, Groningen, NL) while inside the MRI. They performed 30 seconds of exercise at their predicted maximal value, 60 seconds at 85% of maximum, and five bouts of 30-second exercise at 65% of maximum, separated by 15 seconds of rest. All exercise tests were performed on the same day, in the same order for all participants (30 s, 60 s, and 5 × 30 s). Each of these exercise tests were separated by 5 minutes of rest. To determine the resistance to apply to the leg extension ergometer, we calculated target maximal power (wattage) using the Fleisch equation (Equation (3)). We then applied the maximal power for 30 seconds, 85% of this calculated power for 60 seconds, and 65% of this power for the five bouts of exercise. We note that there is a baseline resistance of 5 watts with the Lode MRI cycle, so all predicted and actual power outputs were calculated/measured, and then an additional 5 watts was added.

Maximal Exertion Power (watts) = (Body Mass × 0.45) × 0.4(3)

Spectra were obtained immediately following each exercise bout and in between each bout of the 5 × 30-second exercise. ATP production rates in each of the bioenergetics systems were calculated according to the equations developed by Newcomer and colleagues [32], as previously described by our group [33].

### 2.5. MR Data Analysis

Spectral analysis was performed using Java-based magnetic resonance user interface (jMRUI) (v. 4.0). Areas under inorganic phosphate (Pi), phosphocreatine (PCr), and the three peaks of adenosine triphosphate (ATP; γ, α, β) were calculated with an AMARES (advanced method of accurate, robust, and efficient spectroscopic fitting) algorithm, assuming 100% Lorentzian line shapes for all peaks, as previously described [27]. The concentrations of each peak were normalized to 41.3 mmol, the total sum of muscle phosphate [34]. Changes in intracellular magnesium (Mg^2+^) and pH during exercise were calculated from the chemical shift of β -ATP with respect to PCr and Pi with respect to PCr, respectively [35]. PCr recovery curves were fit with a monoexponential function with tau PCr representing 63% of the metabolite’s recovered value.

### 2.6. Statistical Analyses

Demographic characteristics of the participants were assessed using descriptive statistics (means and standard deviations calculated). The primary purpose of this paper was to examine the feasibility, i.e., whether the exercise MRS protocols could be successfully completed by children post-HSCT [29]. We considered successful execution of the protocol to be that the participants were able to complete the exercise assessments in the MRI, and that the data received were able to be analyzed for relevant variables. To examine whether there were differences in power achieved during MRI exercise protocols, daily physical activity levels, skeletal muscle metabolism, and VO_2_ peak in children post-HSCT vs. healthy controls, we conducted *t*-tests, and statistically significant differences were considered at a *p* < 0.05. Statistical analyses were performed in STATA (11.1, College Station, TX, USA).

## 3. Results

### 3.1. Descriptive Characteristics

Nine patients from the HSCT clinic at The Hospital for Sick Children consented to participate in this study. Two participants had disease-related complications and were excluded from the study, one aged out of the study’s HSCT time limitation prior to testing (allogeneic HSCT > 36 months prior) and one participant did not complete study procedures due to time limitations with our MRI scheduling. Therefore, a total of five children/adolescents post-HSCT and five HCs completed the study procedures for this pilot feasibility study.

Participants’ descriptive characteristics are shown in Table 1. The mean age (± standard deviation) of the HSCT group was 11 ± 1.2 years, and the mean age of the HC group was 12.8 ± 2.4 years (*p* > 0.05). The mean BMI of the HSCT group was 19.2 ± 3.8 kg/m^2^, and the mean BMI of the HC group was 18.3 ± 4.7 kg/m^2^ (*p* > 0.05). Body composition (i.e., body fat percentage and total body lean mass) were not different in the HSCT group vs. the HCgroup (*p* > 0.05). The primary diagnosis requiring HSCT for the children varied; two children had aplastic anemia, and three had leukemia. On average, children were 15.8 ± 5.3 months from their HSCT when they completed study testing.

### 3.2. Aerobic Exercise Capacity and Habitual Daily Physical Activity

Exercise characteristics are presented in Table 2. Children post-HSCT had significantly lower predicted VO_2_ peak compared to the HC group (HSCT: 27.8 ± 3.4 vs. HC: 40.3 ± 8.1 mL kg^−1^ min^−1^, *p* = 0.015). Habitual daily physical activity (hours/day) was similar between the HSCT and HC groups during the week, however, average weekend total activity (hours/day) was lower in the HSCT group, and average weekend total inactivity (hours/day) was higher in the HSCT group vs. the HC group (*p* = 0.006 for both).

### 3.3. Magnetic Resonance Spectroscopy

Participants performed three exercise bouts in the MRI designed to test the three energy systems: 30 seconds of maximal exercise, 60 seconds at 85% of maximum, and five bouts of 30-second exercise at 65% of maximum. All HSCT participants and HCs completed the 30-second exercise MRS testing, and the 5 × 30-second exercise MRS testing. One HSCT and HC participant did not complete the 60 second exercise MRS test, due to time restrictions in the MRI suite.

The mean predicted power (Table 3) was similar between the HSCT participants and HCs for each of the 30-second (12.12 vs. 13.24 watts, respectively; *p* = 0.46), 60-second (11.05 vs. 12.00 watts, respectively; *p* = 0.46), and 5 × 30-second exercise tests (9.63 vs. 10.36 watts, respectively; *p* = 0.47). If pedaling during the exercise bout became too difficult to maintain, wattage was reduced to allow the participants to complete the full exercise time. Therefore, we also recorded the actual power achieved during each exercise bout. The actual power achieved was similar for HSCT partipants and HCs during the 30- and 60-second exercise tests (*p* > 0.05). HSCT and HC partipants were able to maintain between 78–105% of their prescribed power. By the 5 × 30-second test, the actual power achieved was lower in the HSCT participants vs. HCs (6.20 ± 1.30 vs. 9.38 ± 1.38 watts, respectively; *p* = 0.009). HSCT participants maintained 64% of the prescribed power during this bout of exercise compared to HCs who were able to maintain 90% of their prescribed power.

^31^P-MRS results are presented in Table 4. No statistically significant differences were observed between the HSCT and HC groups at rest for Pi, PCr, Pi/PCr, or PCr time constant (*p* > 0.05). No differences were observed in any MRS variables following each of the 30 second, 60 second, or 5 × 30 second exercise protocols (*p* > 0.05 for all).

## 4. Discussion

Exercise intolerance and resulting physical dysfunction are common outcomes in children treated with HSCT [11]. There are many potential factors that contribute to poor physical function, one of which might be impaired skeletal muscle metabolism, leading to inefficiency of the muscle’s ability to engage in exercise. However, no published studies have examined skeletal muscle function at the cellular level in children post-HSCT. As a first step to being able to address this question, we conducted an implementation-focused feasibility study, and found that our MRS exercise protocol in the MRI is feasible in children post-HSCT. All HSCT participants successfully completed the 30-second exercise MRS testing, and the 5 × 30-second exercise MRS testing. One HSCT participant did not complete the 60-second exercise MRS test; however, this was a due to logistics issues and not participant-related complications. Our primary finding is that children post-HSCT are able to complete the MRS protocol and produce analyzable data indicative of skeletal muscle function.

We found that each of the participants in the study was able to complete the majority of the MRS protocol as designed. This was evidenced by the actual power output being similar between the HSCT and HC groups during the 30- and 60-second exercise bouts. However, children post-HSCT were unable to maintain the prescribed power during the 5 × 30-second exercise bout. HSCT participants were only able to maintain 64% of their predicted power (vs. 90% in the HC group; *p* = 0.009) during the 5 × 30 s test. This finding is not surprising, and indicates that children post-HSCT were more fatigued by the time the third exercise test was completed. The ability to maintain physical activity is indeed reduced in the children post HSCT, as they were not able to maintain power during short exercise bouts like their healthy counterparts. This is indicative of the exercise intolerance present in children post-HSCT.

The secondary aim of this study was to examine if there were any differences in exercise capacity, daily physical activity, and skeletal muscle metabolism in children post-HSCT vs. HCs. We found that following a submaximal cycle test, children post-HSCT had significantly lower aerobic capacity compared to HCs (VO_2_ peak: 27 vs. 40 mL kg^−1^ min^−1^ mL/kg min). A mean predicted VO_2_ peak value of 27 mL kg^−1^ min^−1^ indicates poor aerobic capacity; a normal value for children derived from a cycle protocol such as the one used in this study is at least ~36 mL kg^−1^ min^−1^ [36]. Our findings are similar to other studies that report VO_2_ peak in children post-HSCT. For example, one study examined VO_2_ peak determined by an incremental cycle test in 63 children post-HSCT vs. healthy controls, and repoted that VO_2_ peak was 37.4 26 mL kg^−1^ min^−1^ (95% CI: 35.3; 39.5) in the patient group and 44.6 mL kg^−1^ min^−1^ (95% CI: 42.5; 46.8) in the healthy controls [37]. We note that their HSCT participants were on average 7 years (range 3.5–10.4 years) post-HSCT, as their study was a long-term follow-up [37]. In the current study, the median interval from HSCT was much shorter (15 months) and can account for the lower VO_2_ peak values we report. Our values are similar to those of San Juan et al. [38], who reported a pre-training VO_2_ peak of 26 mL kg^−1^ min^−1^ in their HSCT group (*N* = 8; age 10.9 ± 2.8 years, time since transplant between 2–12 months). Overall, our pediatric HSCT recipients demonstrated poor aerobic exercise capacity.

When we examined habitual daily physical activity levels, interestingly, we found that children post-HSCT had similar activity levels compared to the HCs during the week. On the weekend, however, children post-HSCT engaged in less activity (6 vs. 9 hours per day, respectively, *p* = 0.006) and more inactivity (18 vs. 15 hours per day, respectively, *p* = 0.006) compared to the HCs. One hypothesis for this finding is that children post-HSCT maintain weekday activity levels, as they have a scheduled day that involves attending school, and attempting to keep as “normal” as a daily life as possible. However, by the weekend, the children post-HSCT are fatigued and engage in less daily activity, using the weekend to recover. With that said, we interpret these findings with caution, as our study was not powered to detect physiological changes between the HSCT and HC groups.

Unlike aerobic capacity and daily activity level, skeletal muscle metabolism as measured by MRS was not different in children post-HSCT compared to the HCs. Previous studies in other children with chronic diseases who experience exercise intolerance have found that skeletal muscle metabolism is impaired when compared to healthy controls [26,27]. For example, in one study by our research group that used similar methods to the current protocol, girls with Turner syndrome had a greater difference in rest and end-exercise skeletal muscle pH compared to HCs after 30 seconds and 90 seconds of exercise, which is suggestive of greater anaerobic stress during exercise. This may lead to feeling muscle fatigue and pain due to lactic acid build up during high intensity activity [26]. We expected that we might observe similar changes to muscle metabolism in the HSCT group vs. the HC group; however, as previously mentioned, our current study was not powered to detect differences and thus presents a potential for Type II error. In future studies we plan to examine MRS in a larger group of participants powered to detect physiological changes.

There are limitations of the current study. As mentioned above, we only measured MRS variables in five HSCT participants and five HCs. We did not power this study to examine physiological differences in the variables; it was a pilot study to determine if children following HSCT could complete our exercise MRS protocol. Our future direction includes conducting a study with a greater number of participants to accurately determine differences in skeletal muscle function. Another potential limitation is that we matched our HSCT and HC participants based on age and BMI. We did not match children who were pre-pubertal based on sex, because exercise capacity and changes to muscle as a result of hormone differences do not begin until puberty [39]. However, future studies should consider examining/matching participants by Tanner stage. All but one individual in this feasibility study was female; our future larger study should include a representative sample of males.

## 5. Conclusions

In conclusion, the use of exercise protocols in conjunction with MRS to assess peripheral skeletal muscle metabolism is feasible, as indicated by our observations that children post-HSCT had success in completing the MRS protocol and produced analyzable data. As well, we report that children post-HSCT have poor aerobic exercise capacity and engage in less weekend daily physical activity (vs. HCs). The MRS protocol discussed herein can therefore be used in future studies to determine if skeletal muscle function is associated with poor exercise capacity in a larger sample of children post-HSCT.

## Figures and Tables

**Table 1:** Demographic characteristics.

	HSCT *N* = 5	HC *N* = 5	*p*-value
Age (years)	11 ± 1.2	12.8 ± 2.4	0.172
Weight (kg)	40.4 ± 9.0	45.5 ± 15.6	0.541
Body mass index (kg/m^2^)	19.2 ± 3.8	18.3 ± 4.8	0.749
Total body fat tissue (%)	29.1 ± 8.5	23.7 ± 7.1	0.348
Total body lean mass (kg)	28.3 ± 5.5	35.7 ± 11.0	0.226
Time post HSCT (months)	15.8 ± 5.3	N/A	N/A

Mean ± standard deviation unless otherwise indicated. HSCT: haematopoietic stem cell transplant; HC: healthy control.

**Table 2:** Exercise Characteristics.

	HSCT *N* = 5	HC *N* = 5	*p*-value
VO_2_ peak (mL kg^−1^ min^−1^)	27.8 ± 3.4	40.3 ± 8.1	0.015 *
Avg weekday total activity (hours/day)	5.3 ± 1.8	6.6 ± 3.3	0.476
Avg weekday total inactivity (hours/day)	18.7 ± 1.8	17.4 ± 3.3	0.484
Avg weekend total activity (hours/day)	6.0 ± 1.4	9.3 ± 1.4	0.006 *
Avg weekend total inactivity (hours/day)	18 ± 1.4	14.7 ± 1.4	0.006 *

Mean ± standard deviation unless otherwise indicated. * HSCT vs. HC.

**Table 3:** MRI exercise power results.

	HSCT *N* = 5	HC *N* = 5	*p*-value
Power achieved 30 s (watts)	10.25 ± 2.63	13.67 ± 4.04	0.229
% of predicted power	85%	103%	
Power achieved 60 s (watts)	8.63 ± 2.98	12.67 ± 3.06	0.139
% of predicted power	78%	105%	
Power achieved 5 × 30 s (watts)	6.20 ± 1.30	9.38 ± 1.38	0.009
% of predicted power	64%	90%	

MRI: magnetic resonance imaging. Mean ± standard deviation unless otherwise indicated. % of predicted power = mean power achieved/mean predicted power × 100.

**Table 4:** MRS results.

^31^P-MRS Measurement		HSCT *N* = 5	HC *N* = 5
Mean Pi (mM)	rest	2.05 ± 0.32	2.02 ± 0.25
Mean PCr (mM)	rest	19.0 ± 1.33	18.55 ± 0.75
pH dpH	rest 30 s 60 s	6.87 ± 0.12 0.18 ± 0.05 0.27 ± 0.16	6.79 ± 0.21 0.14 ± 0.18 0.15 ± 0.17
Pi:PCr (ratio)	rest 30 s 60 s 5 × 30 s	0.108 ± 0.013 1.11 ± 0.62 1.99 ± 1.15 1.42 ± 1.03	0.108 ± 0.013 1.33 ± 0.814 1.60 ± 0.33 2.18 ± 2.58
PCr time constant (s)	30 s 60 s 5 × 30 s	25.86 ± 10.3 29.69 ± 9.38 24.91 ± 9.51	29.88 ± 13.7 27.67 ± 18.8 28.25 ± 14.3
ATP production rate (mM/s) High energy phospagen	30 s 60 s 5 × 30 s	0.258 ± 0.100 0.173 ± 0.032 0.316 ± 0.128	0.284 ± 0.105 0.168 ± 0.026 0.306 ± 0.117
ATP production rate (mM/s) Anaerobic glycolysis	30 s 60 s 5 × 30 s	0.390 ± 0.078 0.343 ± 0.167 0.552 ± 0.361	0.286 ± 0.520 0.205 ± 0.164 0.416 ± 0.676
ATP production rate (mM/s) Oxidative phosphorylation	30 s 60 s 5 × 30 s	0.264 ± 0.131 0.275 ± 0.056 0.200 ± 0.068	0.246 ± 0.045 0.338 ± 0.068 0.162 ± 0.072

MRS: magnetic resonance spectroscopy. Mean ± standard deviation. *p* > 0.05 between HSCT and healthy controls (HCs) for all variables.
